# Nobody Doesn’t Like Negative Concord

**DOI:** 10.1007/s10936-021-09816-w

**Published:** 2021-11-12

**Authors:** Mora Maldonado, Jennifer Culbertson

**Affiliations:** 1 Centre for Language Evolution, University of Edinburgh, Edinburgh, United Kingdom; 2 Present Address: Departament de Traducció i Ciències del Llenguatge, Universitat Pompeu Fabra, Barcelona, Spain

**Keywords:** Negation, Artificial language learning, Jespersen’s generalization, Negative dependencies

## Abstract

Languages vary with respect to whether sentences with two negative elements give rise to double negation or negative concord meanings. We explore an influential hypothesis about what governs this variation: namely, that whether a language exhibits double negation or negative concord is partly determined by the phonological and syntactic nature of its negative marker (Zeijlstra 2004; Jespersen 1917). For example, one version of this hypothesis argues that languages with affixal negation must be negative concord (Zeijlstra 2008). We use an artificial language learning experiment to investigate whether English speakers are sensitive to the status of the negative marker when learning double negation and negative concord languages. Our findings fail to provide evidence supporting this hypothesised connection. Instead, our results suggest that learners find it easier to learn negative concord languages compared to double negation languages independently of whether the negative marker is an adverb or an affix. This is in line with evidence from natural language acquisition (Thornton et al. 2016).

## Introduction

Languages exhibit a wide range of variation in how negative words interact with one another across contexts. In some languages, each negative expression in a sentence (or clause) necessarily contributes an independent semantic negation. For example, as illustrated in (1-a) and (1-b), in Dutch, both the negative marker ‘niet’ and the negative indefinite ‘niemand’ can independently be used to express sentential negation. But when these negative elements are *combined* in a sentence, as in (2), this leads to a double negation interpretation, which in turn results in a positive meaning.^1^ Languages like Dutch are often referred to as *double negation* languages.

1.
  - Jan rent niet.
    Jan run NEG.
    ‘Jan doesn’t run’
  - Niemand rent.
    N-body run.
    ‘Nobody runs’
2. Niemand rent niet.
  N-body run NEG.
  ‘Nobody doesn’t run’ $\rightarrow$ ‘Everybody runs’

In many other languages, however, the combination of two or more negative expressions can yield a single semantic negation, constituting what is usually known as *negative concord*. Consider the following example from Serbian:

(3) Niko ne trči
  N-body NEG run
  ‘Nobody run’

Despite containing both a negative particle (‘ne’) and a negative existential (‘niko’), the sentence in (3) does not lead to a double negation reading as in (2), but to a single negation interpretation. Languages like Serbian are thus known as *negative concord* languages. While the class of languages that allow negative concord interpretations is far from homogeneous^2^, here we focus on languages like Serbian, which have been categorized as *strictly negative concord* (Giannakidou 1998, 2006). Unlike the indefinite ‘niemand’ in Dutch, negative expressions like ‘niko’ in these languages have the distinguishing property that they cannot generally occur without a negative marker, as exemplified in (4-a). Still, these expressions have negative force on their own, as illustrated by the fact that they can show up in fragmentary answers with a negative meaning (e.g., (4-b)). Words like ‘niko’ in negative concord languages are known as *n-words* or negative concord items (NCIs) (Laka 1990) to differentiate them from the negative quantifiers in double negation languages, like ‘niemand’ in Dutch.

(4)
  *Niko trči.
    *N-body run.
  - Ko trči? Niko.
    who came? N-body
    ‘Who came?’ Nobody.

The contrast between (2) and (3) illustrates the existence of cross-linguistic variation with respect to the interpretation of sentences with multiple negative expressions. A natural question, then, is what determines the distribution of double negation and negative concord interpretations. Debates in the literature have mostly centered on whether negative concord or double negation should be considered the default interpretation for these multiple-negation sentences, and on whether their interpretation might be explained by other linguistic properties (De Swart 2009; Déprez 2011; Zeijlstra 2004; Giannakidou and Zeijlstra 2017).

Evidence from typology and acquisition has been used to support the hypothesis that, despite featuring an apparent violation of compositionality, negative concord is actually more primitive than double negation. For instance, languages that exhibit negative concord seem to be more frequent typologically than those that exhibit double negation (Haspelmath 2013; van der Auwera and Van Alsenoy 2018).^3^ Further, English-speaking children have been shown to assign negative concord interpretations to sentences with two negative expressions, differing from adult (Standard) English speakers, who consistently interpret these sentences as double negation (Thornton et al. 2016). This non-adult-like interpretation has also been documented in (slightly older) German-speaking children (Nicolae and Yatsushiro 2020), suggesting that children might go through a ‘negative concord’ stage regardless of their native language. All this has been taken to suggest that negative concord might be the default or more primitive interpretation for sequences of negative elements.

One traditional counter-argument to this idea comes from the fact that the hypothetical preference for negative concord does not seem to drive language change. In principle, one would expect that if humans had a general bias in favor of negative concord interpretations, languages would tend to change from double negation to negative concord over time. This doesn’t seem to be the case. Instead, the history of Germanic and Romance languages reveals a cyclic pattern of change: double negation becomes negative concord, then double negation arises again, and so on, driven by changes in the form and interpretation of the negative expressions involved (negative markers and indefinites; Jespersen 1917; Zeijlstra 2016; Kiparsky and Condoravdi 2006).

Beyond the question of whether negative concord or double negation is more primitive, the source of cross-linguistic variability in negative interpretations is also debated. Accounts of the difference between double negation and negative concord languages often rely on the existence of other linguistic properties that correlate with the kinds of interpretations that sentences like (2) and (3) can receive. In particular, this cross-linguistic variability has been attributed to differing properties of negative indefinites or of negative markers (Zeijlstra 2004; Déprez 2011; De Swart and Sag 2002; Biberauer and Zeijlstra 2012; Giannakidou and Zeijlstra 2017). Here, we focus on the hypothesized role of the negative marker, and specifically on an observation first made by Jespersen in 1917.

Jespersen (1917) noted that whether a language is double negation or negative concord appears to correlate with the phonological and syntactic nature of the negative marker. Languages which only have a phonologically strong negative marker, like the adverb ‘niet’ in Dutch, exhibit double negation, while languages with phonologically weaker markers, like the particle ‘ne’ in Serbian, are negative concord. This generalization was later weakened by Zeijlstra (2004) to account for the fact that some languages with adverbial negative markers are in fact negative concord (e.g., Quebecois, Déprez 1997). According to Zeijlstra’s reformulation, if a language only has an affix or a particle as negative marker, the language exhibits negative concord. By contrast, languages with negative adverbs are unconstrained in this respect. That is, Zeijlstra’s reformulation only rules out the existence of double negation languages with a phonologically weak affix as negative marker.^4^

Besides weakening Jespersen’s original generalization, Zeijlstra’s reformulation focuses on syntactic rather than phonological properties of the negative marker: in particular, particles and affixes are syntactic heads, whereas adverbs are phrases, e.g., adjoined to the verb phrase, with no associated functional projection (Zeijlstra 2004, 2008; Zanuttini 1991). By virtue of being syntactic heads, negative particles and affixes license agreement with other negative elements, triggering the creation of syntactic dependencies between negative items in the clause. This leads to a negative concord interpretation. Double negation languages whose negative marker is an affix or particle are thus predicted to be impossible by virtue of the syntactic nature of the negative marker.^5^

In this paper, we investigate whether the Jespersen-Zeijlstra generalization (i.e., Zeijlstra’s reformulation of Jespersen’s original generalization) reflects a cognitive constraint on possible languages. We do this by testing whether learners are sensitive to the correlation between the status of the negative marker and the interpretation of a sentence with two negative elements. We expose English-speaking adult learners to one of four miniature artificial languages which vary along these two dimensions: affixal vs. adverbial negative marker and double negation vs. negative concord interpretation. We predict that, if the Jespersen-Zeijlstra generalization reflects a cognitive constraint (or bias), then languages which are ruled out by the generalization should be harder to acquire than those which are not. That is: learners are expected to find it more difficult to learn a double negation language when the negative marker is an affix than when it is an adverb. By contrast, learners are not predicted to differ in their ability to learn negative concord based on the status of the negative marker. Crucially, then, we predict an interaction between marker type and interpretation such that the type of negative marker should have a stronger effect when learning a double negation than a negative concord language, regardless of whether participants have an overall preference for one type of interpretation or negative marker.

In the experiment we report below, we use English-speaking participants. It is thus worth considering, before we continue, what the impact of using an English-speaking population might be. Traditionally, Standard English has been described as a double negation language and distinguished from specific vernacular varieties of English which are known to instantiate negative concord (e.g., Appalachian English; Nevalainen 2006). However, recent experimental results suggest that speakers of Standard English can consistently generate both double negation and negative concord interpretations for sentences with multiple negative expressions (Blanchette 2017; Blanchette et al. 2018; Blanchette 2019).^6^ Some researchers have used this evidence to support the idea that Standard English is in fact an inherently negative concord language and that the apparent preference for double negation is due to sociolinguistic factors (Blanchette 2015); for others, these facts have been instead taken to suggest that Standard English is a double negation language which shows some concord behavior (Zeijlstra 2004). For our purposes, the fact that English speakers vary with respect to how they interpret sentences with multiple negative elements in their native language suggests that they might also vary with respect to which interpretation they assign to these sentences in a new language. Similarly, Standard English exhibits variation with respect to the form of the negative marker. The language has an adverb ‘not’ and a contracted form ‘n’t’, which behaves as an affix (syntactic head; Adger 2003). While the contracted form ‘n’t’ is much more frequent than the longer ‘not’ in adult speech, both markers are acquired by children around the same time (Jasbi et al. 2020). Consequently, English speakers might also vary with respect to the kind of negative marker they find easier to learn in a new language. Taken together, these properties of English suggest that we cannot straightforwardly expect English speaking participants to prefer one specific type of interpretation or marker a priori. What is crucial for our purposes is that participants’ experience with English would not, on its own, lead to an interaction between marker and interpretation of the kind predicted by Jespersen-Zeijlstra’s generalization.^7^

## Methods

### Design

This experiment uses an ease of learning design, in which participants are taught a miniature artificial language, and then tested on how accurately they are able to learn it (see Culbertson 2019, for review of artificial language learning experiments in syntax). As described above, we manipulated two between-subjects factors: Marker and Interpretation. The Marker condition determined whether the negative marker in the miniature language was an affix or an adverb. The Interpretation condition determined whether sentences involving both sentential negation and a negative existential were assigned a double negation (henceforth, DN) or a negative concord (henceforth, NC) meaning. Note that the Interpretation condition also determined the set of sentence types a participant was trained on. This is described in detail in the following section.

### Materials

The input language contained 5 nonce content words (3 verbs and 2 nouns), 2 proper names, 4 quantifiers and one negative marker. All words were created following English phonotactics and were presented orthographically. The three nonce verbs were “jeck”, “rald” and “fudd”, which referred to the events of ‘running’, ‘rotting’ and ‘eating’ respectively. The nouns were “blan” and “affle”, referring to ‘banana’ and ‘apple’. We additionally used the proper names “John” and “Mary”. There were two universal quantifiers (“idho”, “etha”) and two negative existential quantifiers (“midho” and “metha’), which varied in animacy: “idho” and “midho” quantify over human individuals and “etha” and “metha” range over inanimate entities. Quantifiers were similar to each other, following what is found in many natural languages, to facilitate learning.

The negative markers in the artificial language differed depending on the Marker condition: the negative marker was either the affix or an adverb. There are a number of dimensions one could in principle choose from in order to differentiate an affix from an adverb, including phonological weight, boundedness, the possibility of movement, etc. Here we chose to focus on morpho-phonological features. The short affix “im” was orthographically attached to the verb, whereas the longer adverb “imek”, was represented by an independent orthographic word. We kept the position of the two elements constant, which has the advantage of minimizing linear order differences between the groups, which are in principle not at stake here.

Participants were trained on two types of sentences: *simple* and *quantificational*. In both types, word order was Subject-Verb-Object, and negation appeared immediately after the verb. Table 1 illustrates the different sentence configurations for each Interpretation condition.

Simple sentences involved nominal subjects. Each verb form had two possible nominal subjects: the two proper names were used with the verbs corresponding to eating and running events, and the two fruit-denoting nouns with the rotting event. The object in transitive sentences (i.e., involving the eating event) was randomly selected from the fruit-denoting nouns. Simple sentences could be affirmative or negative (Noun-Verb and Noun-Verb-neg in Table 1), and all negative sentences included the negative marker. All participants were exposed to the same set of simple sentences, modulo the type of negative marker.

Quantificational sentences involved universal and negative existential quantificational subjects. The verb dictated the animacy of the quantifier: human-denoting quantifiers appeared with eating and running events, and object-denoting quantifiers with rotting events. All participants were trained on sentences with universal quantificational subjects (Universal-Verb in Table 1). However, unlike simple sentences, the specific configuration of quantificational sentences with negative existential subjects depended on the Interpretation condition. Participants in the DN conditions were trained on two configurations involving negative existentials: Neg-Existential–Verb–neg and Neg-Existential–Verb. By contrast, participants in the NC conditions were only exposed to a single such configuration: Neg-Existential–Verb–neg. This meant that the number of sentence types differed between conditions. To match the number of trials across conditions, the Neg-Existential–Verb configuration used in the set of trials for DN conditions was replaced in NC conditions with Universal–Verb sentences.

Sentences of the type Neg-Existential–Verb–neg were the critical configuration. These were encountered in all conditions, and involve both the negative existential and the negative marker. However, depending on the Interpretation condition, these have either a positive (DN) or a negative (NC) meaning.

Participants were additionally trained on quantifiers in isolation, which were used as one-word, fragment answers to English wh-interrogatives. The interrogative could be ‘Who is running?’, ‘What is rotting?’ or ‘Who is eating a banana/apple?’. The quantifiers then serve to describe a situation where either no character/fruit was running/rotting/eating or all of them were.

The intended meaning of each sentence in the language was conveyed through a picture, as specified in Table 1. Each sentence in the language had two pictures associated with it: a target picture which would make the sentence true, and a foil picture which would make the sentence false. Foil pictures falsified the sentence by featuring a scenario in which the entity or entities denoted by the subject do not satisfy the predicate denoted by the verb.

**Table 1:** Sentence configuration and meaning as a function of the Interpretation condition. The Marker condition, when relevant, is indicated between parentheses

Interpretation	Sentence type		Configuration/Meaning	
Both	simple	Noun–Verb	Noun–Verb–NEG	
		*Mary jeck*	*Mary jeckim (aff)*	
			*Mary jeck imek (adv)*	
	
Double Negation	quantificational	Universal-Verb	Neg-Exist.–Verb	Neg-Exist.–Verb–NEG
		*Idho jeck*	*Midho jeck*	*Midho jeckim (aff)*
				*Midho jeck imek (adv)*
		
Negative Concord	quantificational	Universal-Verb		Neg-Exist.–Verb–NEG
		*Idho jeck*		*Midho jeckim (aff)*
				*Midho jeck imek (adv)*
	

### Procedure

The experiment was implemented using JavaScript and presented to participants in a web browser, with all instructions in English. Participants were told they were going to learn a foreign language called Čelniŝki. There were four experimental phases, and each phase included an *exposure*, a *comprehension* and a *production* task. The experiment proceeded as follows:

*Phase 1: Training and testing on simple affirmative sentences.* Participants were first trained on simple affirmative sentences. In each exposure trial, a simple affirmative sentence appeared in the screen with a corresponding picture (12 trials, 2 per subject-verb pair). Participants were then tested on their ability to comprehend and produce simple affirmative sentences (6 trials per task, 1 per subject-verb pair). On each comprehension trial, participants were instructed to match a sentence in the language with one of two pictures: a target picture, which made the sentence true, and a foil picture, which falsified the sentence. Feedback was provided, indicating whether the response was correct or incorrect and highlighting the correct picture choice. In production trials, participants saw an image, and had to type in a description using the new language. The subject of the sentence was already filled-in in the description field; participants had to complete it. Feedback was provided in the form of the expected answer regardless of the participant’s response.

*Phase 2: Training and testing on simple affirmative and negative sentences.* The basic procedure in this phase was analogous to Phase 1, except that participants were additionally exposed to simple *negative* sentences. To facilitate the learning of the negative marker, simple affirmative and negative sentences were presented in pairs during the exposure block (12 trials, 2 per subject-verb pair). The comprehension block included 12 sentence-picture matching trials featuring negative sentences (2 per subject-verb pair) and 6 featuring affirmative sentences (1 per subject-verb pair). Production trials were also presented in pairs of affirmative and negative sentences. On each trial, two contrasting pictures were displayed; e.g., one where a character is running and one where a character is not running. Participants were given the description for one of the pictures and had to type in a description for the other one. As in Phase 1, the subject of the sentence was already provided, and participants had to complete the description. Participants received feedback for their answers.

Immediately following completion of Phase 2, participants were asked to translate the 5 content words they had learned so far (i.e., 3 verbs and 2 nouns) and were given feedback on their answers.

*Phase 3: Training and testing on quantifiers in isolation.* During exposure (12 trials, 2 per quantifier-verb pair), participants were presented a wh-question in English (see Materials) and a picture corresponding to the answer. In each picture either all or none of the five characters or fruits were running/rotting/eating. After one second, the answer to the question in the novel language was displayed on the screen. The answer always involved a quantifier. Participants were then tested on their ability to comprehend and produce these quantifiers as answers to wh-questions (12 trials per block). These tasks proceeded as in Phases 1 and 2.

*Phase 4: Training and testing on quantificational sentences.* Participants were exposed and then tested on both simple and quantificational sentences. The procedure was similar to Phase 1, but there was no feedback during the comprehension and production tasks. There were 30 exposure trials: 6 for each configuration of simple sentences and quantificational sentences (as determined by the Interpretation condition, see above). The comprehension and production blocks included 48 trials each. There were 24 simple and 24 quantificational sentences per block, including 9 in the critical configuration: Neg-Existential–Verb–neg.

**Fig. 1:**
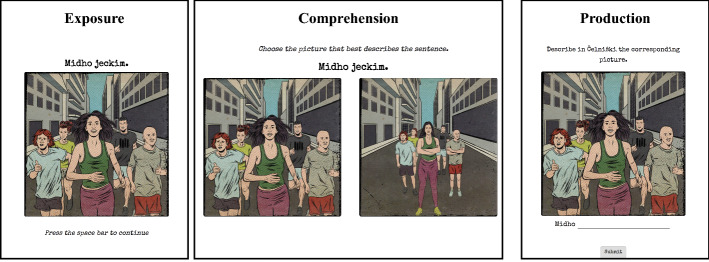
Illustration of Exposure, Comprehension and Production of critical trials in Phase 4. The example corresponds to the Neg-Existential–Verb–neg sentence configuration in the Affix-DN condition

Immediately following completion of Phase 4, participants had to complete a questionnaire about the language they had learnt. First, they were asked to provide a meaning for each of the quantifiers. Then, they were instructed to provide an English translation for three quantificational sentences in the novel language, including a sentence with the critical configuration Neg-Existential–Verb–neg (Fig. 1).

### Participants

A total of 153 English-speaking participants were recruited via Amazon Mechanical Turk. All participants had U.S. based IP addresses. Per our preregistration, participants (N=29) whose accuracy was below 75% in any production or comprehension task across phases 2 and 3 were excluded. The data of the remaining 124 participants was analyzed (Adv-DN: 29, Affix-DN: 38, Adv-NC: 32, Affix-NC: 30). Participants were paid 5 USD for their participation which lasted approximately 25 minutes.

## Results

Recall that, based on Jespersen-Zeijstra’s generalization, we predict that English-speaking learners will find it easier to learn a double negation language when the negative marker is an adverb than when it is an affix; according to this generalization, languages with affixal negation are necessarily negative concord. For learners in the negative concord condition, we did not predict an effect of marker type; negative concord is possible with either type of negative marker. This should lead to an interaction between Marker and Interpretation in how well participants were able to use sentences with two negative expressions.

Following our pre-registration, we analyzed accuracy rates in the comprehension and production of the Neg-Existential–Verb–neg sentence configuration (Phase 4).^8^ We fit separate models for comprehension and production data, but the analysis pipeline was the same. All analyses were carried out in the R programming language and environment (R Core Team 2018), using the lme4 software package (Bates et al. 2007). Responses were analyzed by modeling response-type likelihood using logit mixed-effect models. Fixed effects were Marker, Interpretation and their interaction. Models included random intercepts for subjects and items when possible^9^. Interpretation and Marker predictors were sum-coded. P-values were obtained by a $\chi ^{2}$ likelihood ratio test comparing the full model with a simpler one in which the relevant predictor (i.e., main effect or interaction) was removed. The likelihood ratio test indicates whether or not the full model provides a better fit to the data than the reduced alternative.

### Comprehension

Responses in comprehension trials (i.e., sentence-picture matching trials) were coded as 1 if the picture selected corresponded to the target image, and as 0 otherwise. Per our pre-registration, comprehension trials whose response times were below 500 milliseconds or above 15000 milliseconds were excluded from the analysis. This led to the exclusion of approximately 3% of the critical trials.

**Fig. 2:**
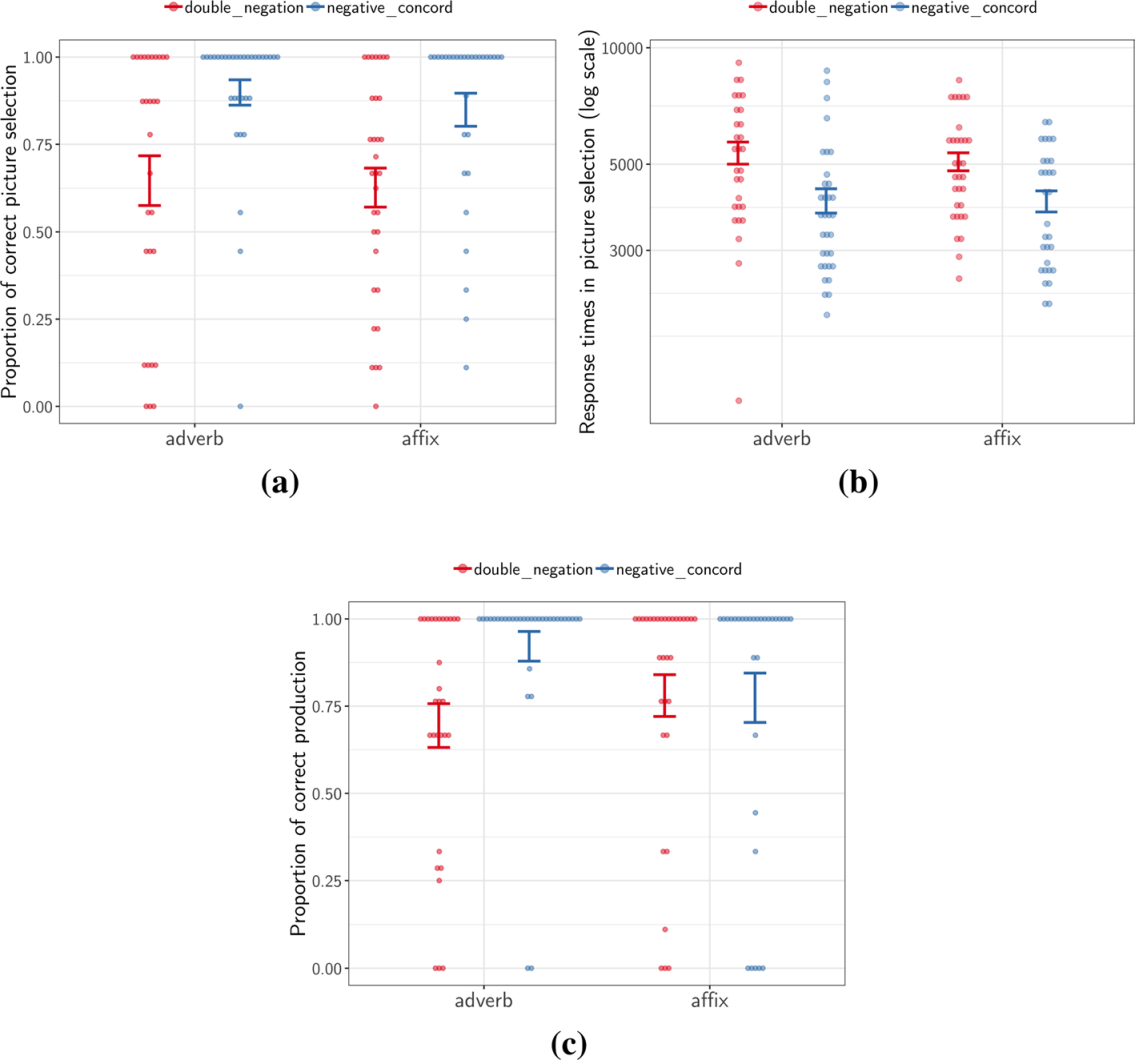
Performance in Neg-Existential–Verb–neg per Interpretation and Marker conditions. **a** Accuracy rates in sentence-picture matching trials (Comprehension task). **b** Log-transformed response times in correct sentence-picture matching trials (Comprehension task). **c** Accuracy rates in picture description (Production task). Error bars represent standard error on by-participant means; dots represent individual participant means

Figure 2a shows the mean proportion of correct picture selection for the critical configuration Neg-Existential-Verb-neg. Model comparison revealed that, contrary to our predictions, the interaction between Marker and Interpretation was not statistically significant ($p=0.91$, $\chi ^{2}<1$). An analysis of the main effect of Interpretation indicated that the proportion of correct responses was significantly higher in the negative concord conditions than in the double negation ones ($p<.001$, $\chi ^{2}=23.4$). There was no main effect of Marker ($p=0.51$, $\chi ^{2}<1$).

As an exploratory analysis, we additionally analyzed the response times corresponding to correct responses during the comprehension task. That is, the time taken to accurately select the target picture. Figure 2b shows mean response times for comprehension trials in the Neg-Existential-Verb-neg configuration. We ran a linear mixed-effects regression model predicting log-transformed response times by Marker, Interpretation and their interaction, and including random intercepts per subject and item. As before, likelihood ratio tests were used to obtain p-values. We found that participants were significantly slower to assign a double negation interpretation than a negative concord one (Main effect of Interpretation: $p<.001$, $\chi ^{2}=27.8$). No significant effect of Marker ($p=.53$, $\chi ^{2}<1$) nor Marker $\times$ Interpretation interaction ($p=0.89$, $\chi ^{2}<1$) were detected.

### Production

In production trials, participants were asked to provide a picture description using the novel language. We only analyzed production trials where participants were expected to produce sentences involving two negative expressions (a negative indefinite and a negative marker). Crucially, the picture that participants had to describe depended on the Interpretation condition, as indicated in Table 1: participants in the DN conditions had to describe a picture where all the fruits or characters were running/rotting/eating, while participants in the NC conditions described a picture where none of the fruits or characters satisfied the predicate.

Responses in these production trials were coded as 1 if the negative marker was present in the description, and 0 if it was absent. Per our pre-registration, we excluded from the analyses responses where: (a) the average Levenshtein distance between the expected and the actual response was greater than 2; or (b) the negative marker was produced in the wrong position. This led to the exclusion of approximately 7% of the critical trials.

Figure 2c shows the proportion of responses where participants correctly produced Neg-Existential–Verb–neg sentences in critical trials. The results of the model comparison revealed that the interaction between Marker and Interpretation was not significant ($p=.14$, $\chi ^{2}=2.09$). However, there was again main effect of Interpretation, though this was marginal ( $p=.057$, $\chi ^{2}=3.61$). This effect indicates that participants were more likely to correctly produce two negative expressions in NC groups than in DN groups. In other words, participants found it easier to produce a sentence with two negative elements when describing a picture were no one is running than when describing a picture where everyone is running. The main effect of Marker was not significant ($p=.63$, $\chi ^{2}<1$).

## Discussion

The Jespersen-Zeijstra generalization suggests an intrinsic connection between the properties of the negative marker in a given language and whether this language is double negation or negative concord. Here we reported the results of an artificial language learning experiment aimed at assessing whether this generalization is grounded in a cognitive constraint or bias active during learning. Our experiment tested whether adult English speakers are sensitive to the status of the negative marker—as a phonologically stronger adverb-like element or an affix—when learning double negation or negative concord languages. We predicted that the status of the negative marker should have a stronger effect when participants were acquiring a double negation than a negative concord language. Specifically, the combination of double negation with an affixal negative marker was predicted to cause learners particularly difficulty.

Contrary to our predictions, we found no evidence for a connection between interpretation and negative marker. Participants showed no sensitivity to the status of the negative marker nor to its impact on interpretation. Instead, we found a consistent main effect of interpretation on learnability: participants found it more difficult to learn a double negation than a negative concord language, regardless of whether the negative marker was adverb-like or affixal.

### The Relative Disadvantage of Double Negation

The relative ease of acquiring a negative concord language was found in both the comprehension and production tasks in our experiment, but this effect was stronger in comprehension. In comprehension, learners were required to interpret sequences of negative expressions. Our results suggest that they found it more difficult to treat each negative element as contributing an independent semantic negation, preferring instead to assign a negative meaning to the whole sentence. This difficulty is attested both in accuracy rates (i.e., percentage of correct responses in the sentence-picture matching task) and in response times, which can be taken to be a more direct reflection of processing. There are a number of explanations which could account for this asymmetry between double negation and negative concord interpretations. First we will entertain explanations that derive from participants’ native language experience.

As mentioned above, English speakers vary with respect to how they interpret sentences with multiple negative elements, and it has been argued that Standard English should be treated as an underlyingly negative concord language (Blanchette 2013, 2015). The bias for negative concord interpretations in our experiment might then be the result of a native language bias: English speakers find it easier to learn a new language which has negative concord because their own native language is also negative concord (at some level).

In order to establish whether a native language bias plays a role in our experiment, it’s crucial to determine participants’ preferred interpretation of English sentences with multiple negative elements (e.g., ‘Nobody is not running’). Unfortunately, given that our pre-registered hypothesis does not predict an effect of interpretation, we did not gather this information. That said, there is certainly an argument to be made against this explanation. Standard English speakers have been consistently shown to prefer double negation interpretations of sentences featuring the configuration used in our study, which consists of a negative indefinite in subject position preceding the negative marker (Blanchette 2017; Blanchette et al. 2018; Blanchette and Lukyanenko 2019). Assuming that our participants belong to the same population as participants in previous studies, we might expect their native language preferences to be similar. By contrast, the preferred interpretation for these types of sentences during learning of the artificial language was negative concord.

Another consideration is that contexts in which double negation interpretations are appropriate tend to be quite restricted, even in languages categorized as strictly double negation (see Footnote 1). For example, a sentence like “Nobody is not running” in its double negation reading might be felicitous as a response to a statement like “Is everyone running? I thought I saw some people just standing around”, but it is less felicitous out of the blue. One result of this is that double negation sentences tend to be quite rare in languages that allow them (while negative concord sentences would be relatively common in a negative concord language). In our experiment, we prioritized similarity across conditions, such that they differed only on our manipulated variables, Marker and Interpretation. We did not manipulate frequencies of double negation or negative concord interpretations across condition, and we did not provide participants with a specialised context for double negation. However, these choices may have contributed to the asymmetry between conditions we found.^10^

As in comprehension, the apparent dispreference for double negative in production might also be explained by native language transfer, or to the lack of context facilitating double negation. However, it is also possible that an aspect of the production task design may have contributed to this effect as well. Recall that, in critical production trials, participants were provided with a negative indefinite and had to complete the sentence. Our results indicated that participants were more likely to produce the negative marker when the overall meaning was negative than when the resulting meaning was positive. The sentence completion aspect of the design may have led participants to ignore the negative indefinite provided, simply producing a negative marker whenever the sentence is negative, e.g., whenever they have to describe a situation where there is no running happening, and to ignore it otherwise.

In addition to the possible explanations outlined above, there are also more general explanations, which may be at play in both comprehension and production. One possibility is that, as has been proposed in the acquisition literature, negative concord is treated as a default by learners (e.g., as a default parameter setting, Nicolae and Yatsushiro 2020). This proposal is based on data from natural language acquisition, which suggests that children consistently interpret sentences with multiple negative words as conveying a single negation, regardless of their native language (Thornton et al. 2016; Nicolae and Yatsushiro 2020). Our experimental results would constitute an extension of this idea to adults learning an artificial language.

Another related explanation lies in the cost associated with processing sentential negation. Psycholinguistic research has repeatedly shown that negative sentences are harder to process than their positive counterparts (see Tian and Breheny 2019, for review). While there are different accounts of this difference in cognitive effort, it may be that double negation interpretations impose additional processing cost simply because they involve processing two negative operators instead of one. The cost associated with processing negation might actually prompt learners to assign a negative meaning to a sentence at the first negative element they encounter. If participants are using this strategy, then negative concord readings will of course be favored. Interestingly, this could in principle explain the observation that children generally prefer negative concord interpretations, without relying on any notion of default parameter settings.

### Lack of Evidence for the Jespersen-Zeijlstra Generalization

None of these explanations of participants’ dispreference for double negation explains straightforwardly why they showed no sensitivity to the type of negative marker. In other words, the question of why we found no evidence confirming the Jespersen-Zeijlstra generalization remains open. Here we outline several possibilities, all of which we hope to address in future research.

Starting again with explanations which rely on participants’ native language knowledge, the impact of the marker status in double negation conditions could in principle be somehow masked by English speakers’ preference for post-verbal affixal negation in the experiment. This would stem from the fact that (as noted in the Introduction) the contracted form ‘n’t’ is more frequent in English adult speech than the longer adverb ‘not’. While there is no evidence that ‘n’t’ is easier to learn than ‘not’ for English-speaking children, nevertheless English-speaking participants might bring an expectation of post-verbal affixal negation to the task. This might in turn result in affixal negation being easier to learn. The relative difficulty of learning a double negation language with affixal negation (predicted by the Jespersen-Zeijlstra generalization) might then be masked by an overall preference for post-verbal affixal negation. Notably however, we have no clear independent evidence for this explanation. For example, we do not see any preference for affixal negation in the negative concord conditions.

A more compelling possibility is that our experiment is not detecting the hypothesized correlation because the artificial languages are not encoding the pertinent distinction between negative markers. Recall that in this study, we manipulated the orthographic properties of two post-verbal negative markers: a longer independent word (‘imek’) and a shorter affix (‘-im’), which appeared attached to the verbal stem. One might question whether these orthographic differences between markers provide a strong enough cue for learners to treat them as morpho-phonologically distinct. Perhaps if the difference in phonological weight was even bigger, or if the experiment were presented auditorily, these differences would trigger the predicted effect.^11^

More importantly, as discussed above, morpho-phonological features are not necessarily sufficient to trigger the syntactic difference on which Zeijlstra’s theory is based. According to this theory, whether a language is double negation depends on whether it has a negative marker that is a syntactic head. Arguably, if learners in our experiment had more evidence to categorize the marker syntactically (e.g., evidence of movement of the adverbial marker), an interaction between marker and interpretation would have emerged.

Lastly, it is of course possible that the Jespersen-Zeijlstra generalization does not reflect a cognitive constraint or bias, and should instead be seen as an accidental regularity, perhaps the product of relying on a rather small typological sample. It has been argued, for example, that cross-linguistic variation in the interpretation of sentences with multiple negative elements is actually modulated by the properties of the negative concord items or of negative indefinites, rather than by the properties of the negative marker (Déprez 2011; Déprez et al. 2015; but see also Biberauer and Zeijlstra 2012 and Giannakidou and Zeijlstra (2017).

## Conclusion

In this paper, we investigated a well-known typological generalization on the distribution and interpretation of negative words: whether a language exhibits double negation or negative concord correlates with the phonological and syntactic nature of its negative marker (Zeijlstra 2004; Jespersen 1917). More specifically, while languages with adverbial negation can exhibit negative concord or double negation, if a language features affixal negation, then only negative concord is possible (Zeijlstra 2008). We ran an artificial language learning experiment to assess whether learners are sensitive to this connection between negative marker and interpretation. We failed to find any evidence to suggest that this generalization has a simple correlate in learning. Instead, our findings reveal that negative concord languages are easier to learn compared to double negation languages, independently of whether the negative marker is an adverb or an affix.

## Footnotes

1 While doubly-negated sentences like (2) are typically marked when uttered out of the blue, they are felicitous in several contexts, including contexts of explicit denial (i.e., when the negative proposition has been already introduced in the common ground) (Fălăuş and Nicolae 2016; Déprez et al. 2015; De Swart 2020).

2 Languages may differ on whether the negative marker in sentences like (3) is mandatory (e.g., Serbian vs. Welsh), and on whether negative concord items like ‘niko’ in pre-verbal position need to be accompanied by a negative marker (e.g., Serbian vs. Spanish).

3 It’s worth pointing out that the majority of languages in these typological samples do not feature either double negation or negative concord, as they lack proper negative indefinites (van der Auwera and Van Alsenoy 2018). In these languages, the negative existential meaning is expressed by combining a sentential negation and a neutral indefinite.

4 This reformulation is also consistent with the acquisition results mentioned above. Standard German, for example, has an adverbial negative marker. While Zeijlstra’s account doesn’t itself explain why German-speaking children would prefer a negative concord reading, it is compatible with the language being either negative concord or double negation. The possibility of both options under this account thus leaves room for this variability in interpretation of two-negation sentences (Nicolae and Yatsushiro 2020).

5 In Zeijlstra’s theory, the existence of negative concord languages with adverbial negative markers (e.g., Quebecois) is explained by positing that the agreement between negative elements may be licensed by a covert element (i.e., phonologically null). A language like Quebecois would still have a negative marker that is a syntactic head, but this marker would be silent. It’s worth noting that this assumption allows Zeijlstra to explain not only Jespersen-Zeijlstra generalization but also the difference between strict and non-strict negative concord (see Zeijlstra 2008, for more details).

6 More specifically, Standard English speakers have been shown to accept both NC and DN readings in their language, as a function of the syntactic, pragmatic and intonational properties of the relevant sentence. For example, adult English speakers are more likely to accept NC interpretations for sentences where the negative marker scopes above the negative indefinite (e.g., ‘John didn’t call nobody’) than for sentences where the negative indefinite has wide scope (e.g., ‘Nobody didn’t call’) (Blanchette 2019).

7 All experimental hypotheses, predictions, and analyses, were preregistered. The complete set of materials and the scripts can be found here.

8 Detailed results for simple and other quantificational sentences can be found in the associated OSF repository. The repository also contains data regarding the translations provided by participants for each of the words in the artificial language, as well as for critical sentences.

9 A maximal random structure (Barr et al. 2013) should also include random by-item slopes. Due to lack of convergence, we could not include random slopes. Similarly, random by-item intercepts had to be removed from the comprehension model.

10 For example, an anonymous reviewer suggests that merely having double negation interpretations be very frequent might be surprising for learners, and might encourage them to mistake these for negative concord sentences.

11 A reviewer suggests that using a post-verbal affix might be problematic, and perhaps the contrast between a post-verbal adverb and a pre-verbal affix would have been more salient. While we agree that this would be interesting to try, there are a few reasons why we did not use this configuration. For example, English speakers might treat a prefixal negative marker as they treat negative prefixes in their language (e.g., ‘im-possible’). This would be problematic, since negative prefixes in English do not trigger sentential negation, and are incompatible with negative concord interpretations. That said, we hope to increase the salience of the morphophonological or syntactic differences between the markers in future work.
